# Annotations

**Published:** 1910-03-12

**Authors:** 


### Annotations.

### The Order of St. John.

The *London Gazette* of March 4 contains a long list of honours and promotions sanctioned by his Majesty in the Grand Priory of the Order of St. John of Jerusalem in England. Most of these are of general rather than professional interest, but there are a few which have been conferred upon medical men. The new Knight of Justice selected from the Knights of Grace is a layman, and inspection of the list reveals that the profession is represented in this highest rank of the Order by two surgeons only out of a total of fifty. There are twelve new Knights of Grace, three of whom are on the Medical Register. These three are Dr. R. A. Gibbons, one of the best-known, successful, and most respected general practitioners in London, from whose pen an instructive clinic will appear in The Hospital very shortly; Lieut.-Col. F. H. Appleby, M.R.C.S., of Newark, a veteran worker both for the Order and for the Auxiliary Forces of the Crown; and Major R. J. Blackham, R.A.M.C., whose promotion also is well deserved. Three more medical men are appointed to be Esquires: these are Major C. A. Hodgetts, of Canada; Dr. C. J. Copp, the local honorary secretary of the St. John's Ambulance Association in the same country, and Major H. B. Yates. The claims of Canadian workers, it may be added, to representation upon the Chapter are recognised in the selection of Lord Strathcona to be a Knight of Grace.

### The Storage of Poisons.

The Executive Committee of the General Medical Council, at a meeting on February 21, had before them the facts concerning the death of a woman from strychnine poisoning, which were elicited at the Coroner's Court at Fulham on January 4, and referred to in an article on "The Storage of Poisons" which appeared in The Hospital of January 15. The attention of the committee was drawn to this matter by a letter from the Clerk to the Privy Council, in the course of which appears the following paragraph:—"I am to enclose a copy of the Order of Council of the 5th June, 1902, approving regulations made by the Pharmaceutical Society of Great Britain for the keeping, dispensing, and selling of poisons within the meaning of the Pharmacy Acts, and to request that you will move the General Medical Council to favour his Lordship with any observations they may have to make on the suggestion that the regulations in question should be extended —by legislation—to medical men." The executive committee resolved to inform the Lord President that the suggestion that the regulations in question should be extended by legislation to medical men had been laid before them, and that they approved of such extension as tending to ensure the safety of the public. The regulations embodied in the Order in Council referred to were summarised in The Hospital in the article to which reference is made above, and it was pointed out that, although medical men are not legally compelled to comply with these rules, they are binding upon pharmacists. Presumably the Privy Council intends that a Bill shall be introduced into Parliament to extend the regulations to medical practitioners who dispense. The regulations are not of an irksome character, and to comply with them should cause little or no inconvenience to practitioners.

### Sleeping Sickness.

The Sleeping Sickness Commission, despatched in 1908 by the Imperial Government under the direction of the Royal Society, is still actively engaged on the shores of Lake Victoria Nyanza. Sir David Bruce, who has been the head of the Commission, has now returned to England *viâ* the Nile, accompanied by Lady Bruce, whose devotion to scientific research is well known to rival that of her distinguished husband. Much has been already accomplished, and matters seem to be well in train for further achievement. In one respect the work to be done has been rendered more difficult than was at first supposed, for Dr. Klein, whose researches have been conducted in the adjacent territory of German East Africa, has proved that the infected *Glossina* retains its parasites and its powers of transferring them to man for a much longer period than the forty-eight hours, which was previously regarded as the limit. It follows from this that a zone which has been cleared of its inhabitants cannot be repopulated without risk of a fresh epidemic until may months have elapsed, and this is a serious difficulty in view of the extraordinary rapidity with which the jungle covers clearings and other reclaimed plots of land in a tropical climate such as that of Uganda. On the other hand, the energetic measures which have been taken do really appear to be promising of success, for no new cases are being reported in Uganda. The camps are, however, full of moribund and hopeless patients, whose death apparently nothing can avert. In certain districts not twenty per cent. of the inhabitants have escaped the ravages of this pestilence; these regions, which comprise either islands or strips along the lake shore, are in consequence much easier to depopulate, and investigation is now being carried out by the members of the Commission to determine for how long the tsetse flies there remain infective. It is understood also that a great deal of new work has been done on the life cycle of the trypanosome in the body of the fly, and this is now in course of publication in the Journal of the *Royal Army Medical Corps*, beginning in the February number. The congratulations of everyone interested in tropical medicine and hygiene will be extended to both Sir David and Lady Bruce on the safe conclusion of their trip.

